# Post-extubation stridor in Respiratory Syncytial Virus bronchiolitis: Is there a role for prophylactic dexamethasone?

**DOI:** 10.1371/journal.pone.0172096

**Published:** 2017-02-16

**Authors:** Esther S. Veldhoen, Charlotte A. Smulders, Teus H. Kappen, Job C. Calis, Job van Woensel, Paulien A. M. Raymakers-Janssen, Louis J. Bont, Marije P. Hennus

**Affiliations:** 1 Department of Pediatric Intensive Care, Wilhelmina Children’s Hospital, University Medical Center Utrecht, Utrecht, the Netherlands; 2 Department of Anesthesiology, Vanderbilt University Medical Center, Nashville, Tennessee, United States of America; 3 Department of Pediatric Intensive Care, Emma Children’s Hospital, Academic Medical Center, Amsterdam, the Netherlands; 4 Department of Pediatric Infectious Diseases, Wilhelmina Children’s Hospital, University Medical Center Utrecht, Utrecht, the Netherlands; National Yang-Ming University, TAIWAN

## Abstract

**Aim:**

The purpose of this study was to determine the incidence of reintubation due to upper airway obstruction in a homogeneous group of ventilated infants with Respiratory Syncytial Virus bronchiolitis. Our secondary objective was to determine whether prophylactic administration of dexamethasone prior to extubation was associated with decreased risk of reintubation.

**Methods:**

This retrospective observational study in two Pediatric Intensive Care Units in 2 university hospitals in The Netherlands included two hundred patients younger than 13 months admitted with respiratory insufficiency caused by Respiratory Syncytial Virus bronchiolitis, requiring invasive mechanical ventilation. A logistic regression analysis with propensity score method was used to adjust for possible confounding.

**Results:**

Reintubation due to post-extubation stridor occurred in 17 (8.5%) of 200 patients. After propensity score matching, administration of dexamethasone prior to extubation was associated with a significantly (p = 0.0011) decreased risk of reintubation due to post-extubation stridor compared to patients not receiving prophylactic dexamethasone (absolute risk reduction 13%, 95% CI 5.3–21%).

**Conclusion:**

Reintubation due to post-extubation stridor is an important complication of ventilation for Respiratory Syncytial Virus bronchiolitis. Dexamethasone administered prior to extubation probably reduces the risk of post-extubation stridor necessitating reintubation in these infants. The results of this study support initiation of a placebo-controlled trial to confirm the beneficial effect of prophylactic dexamethasone.

## Introduction

Respiratory Syncytial Virus (RSV) is the most common cause of seasonal acute respiratory tract infections in children and accounts for more than 80% of lower respiratory tract infections (LRTI) in infants [1–3]. RSV-related hospitalization is required in about 1 to 3% of infected children [4–7]. Unfortunately, as specific treatment for RSV infection is lacking, the most effective therapy remains supportive care [3]. In approximately 10% of hospitalized RSV-infected patients, almost exclusively infants, invasive mechanical ventilation is required because of severe respiratory failure [4,8]. Overall, mean duration of mechanical ventilation is approximately seven days [9].

Even though they are life-saving, intubation and subsequent invasive mechanical ventilation are associated with complications of the upper airway. Post-extubation stridor, persistent subglottic stenosis, and vocal cord paralysis are the most important upper airway complications [6,10–13]. These complications are the result of either reactive laryngotracheal edema of the mucosa, or direct tissue pressure and irritation from the endotracheal tube. Upper airway complications are the main cause for extubation failure (EF) [14,15]. When reintubation is required, patients are increasingly exposed to the hazards of intubation and mechanical ventilation. Reintubated patients have a prolonged duration of ventilation, an increased length of stay in the ICU and an increased mortality rate as compared to patients successfully extubated on the first attempt [9,11,12,16–18]. The overall incidence of EF in all mechanically ventilated children is approximately 5–20% [16–18]. One small study suggests that the EF rate is higher (15%) in infants with a viral bronchiolitis [9].

Prophylactically administered corticosteroids (i.e. given prior to extubation) are believed to reduce the inflammatory response, decrease laryngeal edema, and prevent reintubation [12,19]. The corticosteroid of choice in neonates and children is dexamethasone [20–22]. Meta-analyses report lower reintubation rates in neonates, children and adults that received corticosteroids [12,23,24]. However, trials within the pediatric population are small, and only show a trend towards a reduction in reintubation rates.

We hypothesized a high incidence of post-extubation stridor in RSV-infected infants due to young age and subsequent small airway diameter [21,24], relatively long duration of mechanical ventilation [14] and upper airway mucosal inflammation at time of extubation [13]. When this hypothesis is true, RSV-infected infants may thus have a greater beneficial effect of prophylactically administered corticosteroids. The aim of this retrospective study was to determine the incidence of reintubation due to upper airway obstruction in a homogeneous group of ventilated infants with RSV LRTI. Our secondary objective was to determine whether prophylactic administration of dexamethasone prior to extubation was associated with decreased risk of reintubation in this homogeneous group of ventilated infants with RSV LRTI.

## Materials and methods

### Study participants

All invasively mechanically ventilated patients up to and including 12 months of age with a proven RSV LRTI were eligible for inclusion in this retrospective multi-center observational cohort study. Data were collected at the PICUs of the Wilhelmina Children’s hospital (University Medical Center Utrecht (UMCU), Utrecht, The Netherlands) and of the Emma Children’s hospital (Academic Medical Center, (AMC), Amsterdam, The Netherlands). Both are 14-bed general PICUs. All patients admitted at the PICU for suspected RSV LRTI between November 2008 and April 2014 were considered eligible for inclusion. Suspected RSV LRTI was based on clinical manifestations in the RSV season. Proven RSV infection was based on clinical manifestations of LRTI in combination with a positive nasopharyngeal PCR and/or enzyme immunoassay. In case of inconsistent test results, PCR was considered to be conclusive. Patients were excluded when corticosteroids were administered for other reasons and when patients had pre-existent upper airway pathology. Patients were only included during their first ventilation period, and not after reintubation. Ethical approval was deemed exemption by the institutional review board.

### Data collection and outcome measures

The following variables were considered as possible confounders: age and weight on admission, gender, gestational age, chronic lung disease, congenital heart disease, Down syndrome, previous intubations during surgery or previous ICU admission, palivizumab prophylaxis, traumatic initial intubation, endotracheal tube size, use of a cuffed tube, tube replacement before the first extubation, location of initial intubation, ventilation days. Congenital heart disease was defined as congenital heart disease with hemodynamic consequences requiring surgery. Initial intubation was considered to be traumatic when intubation was recorded as being “difficult” or when several attempts were necessary to intubate. The location of the initial intubation could be either a district general hospital or one of the participating children’s hospitals. Additional data were collected on: presence of post-extubation stridor, treatment given for post-extubation stridor (intravenously administered dexamethasone, nebulized Pulmicort and/or Adrenaline, non-invasive ventilation).

The primary prespecified outcome measure was defined as the necessity for an endotracheal tube because of post-extubation stridor within 24-hours following extubation. Presence of stridor was assessed by a qualified PICU nurse and at least two physicians. No scoring system was routinely used to objectify the stridor. Laryngotracheoscopy was only performed after failure of second extubation attempt or when there was doubt of upper airway obstruction being caused by laryngeal edema.

### Statistical analysis

Analysis was performed under the intention-to-treat principle. All statistical analyses were performed in R software, version 3.2.2 (The R Foundation for Statistical Computing, Vienna, Austria). Statistical significance was defined as a two-sided alpha of 0.05. Continuous variables were visually assessed for a normal distribution using histograms and QQ-plots. Parametric variables were expressed as means with standard deviations, nonparametric variables were expressed as medians with interquartile ranges, and discrete variables were expressed as numbers with percentages.

As the number of outcome events was low in the study population, whereas exposure (administration of prophylactic dexamethasone) was relatively common, a propensity score method was used to adjust for possible confounding. The propensity score is a method to estimate the conditional probability of receiving dexamethasone [25]. Based on prior knowledge, all variables in Table 1 were considered to be potential confounders, except for gender and the location of the initial intubation, as we did not expect these variables to influence the decision to give prophylactic dexamethasone. The propensity score model was initiated by adding all possible confounders to a logistic regression model with prophylactic dexamethasone administration as the dependent variable. All continuous variables were log-transformed because of a skewed distribution. Variables with a very low event rate were excluded from the propensity score model. The participating center was added to the propensity score model, as it was a possible risk factor of the outcome, rather than a true confounder. Based on prior knowledge, 2-way and 3-way interactions with weight and age were considered for endotracheal tube size, prematurity, use of a cuffed endotracheal tube, traumatic intubation, and endotracheal tube replacement. The rationale behind this 3-way interaction was that these variables would depend on both the age of the patient and its weight relative to its age. Improvement of the propensity score model by adding variables and interactions was evaluated based on the change in balance achieved by matching on the propensity score. This balance was assessed using the absolute standardized differences for confounders and risk factors between the study groups after matching. Nearest neighbor matching with a 1:3 ratio was then performed using the logit of the propensity score model as the matching variable, aimed at estimating the average treatment effect (*Match*, *Matching* package, R software). The matching was performed with caliper widths 0.10, 0.20, 0.30 and 0.40 to find the optimal balance [26,27].

**Table 1:** Baseline characteristics of patients receiving dexamethasone prior to extubation or not, before and after propensity score matching.

	Baseline Table: Before Matching		Baseline Table: After Matching^a^	
	Dexamethasone (n = 99)	No Dexamethasone (n = 101)	Dexamethasone (n = 173)^b^	No Dexamethasone (n = 173)^b^
Female sex, n (%)	43 (43)	43 (42)	71 (41)	78 (45)
Weight in kg, median (IQR)	4.2 (3.7–5.0)	4.4 (3.6–5.3)	4.2 (3.6–5.0)	4.2 (3.5–5.1)
Age in days, median (IQR)	38 (29–63)	47 (29–86)	37 (28–61)	38.0 (26–62)
Down syndrome, n (%)	2 (2)	2 (2)	3 (2)	1 (1)
Congenital heart disease, n (%)	2 (2)	2 (2)	4 (3)	2 (1)
Chronic lung disease, n (%)	1 (1)	0 (0)	0 (0)	0 (0)
Prematurity, n (%)	27 (27)	20 (20)	38 (22)	38 (22)
Palivizumab immunization, n (%)	2 (2)	5 (5)	2 (1)	4 (2)
Endotracheal tube size: 3, n (%)	25 (25)	15 (15)	32 (19)	29 (17)
Endotracheal tube size: 3.5, n (%)	62 (63)	69 (68)	125 (73)	128 (74)
Endotracheal tube size: 4, n (%)	10 (10)	14 (14)	16 (9)	16 (9)
Endotracheal tube size: 4.5, n (%)	2 (2)	3 (3)	0 (0)	0 (0)
Cuffed endotracheal tube, n (%)	27 (27)	30 (30)	51 (29)	48 (28)
Previous intubations, n (%)	4 (4)	8 (8)	6 (4)	7 (4)
Endotracheal tube replacement (%)	44 (44)	29 (29)	60 (35)	63 (37)
Traumatic intubation, n (%)	13 (13)	7 (7)	22 (13)	23 (13)
Duration of ventilation in days, median (IQR)	7.4 (5.1–9.5)	5.8 (4.3–8.7)	6.8 (4.7–8.7)	7.0 (4.6–9.4)
Initial intubation in district general hospital, n (%)	87 (88)	81 (80)	150 (87)	141 (81)

IQR = Interquartile Range. The unweighted number of cases in the matched sample was n = 534.

^a^ Cases were matched on the logit of the propensity score, 3:1 nearest neighbor, with a 0.30 caliper

^b^ n = 173 is the weighted number of cases in the matched sample

## Results

**Fig 1:**
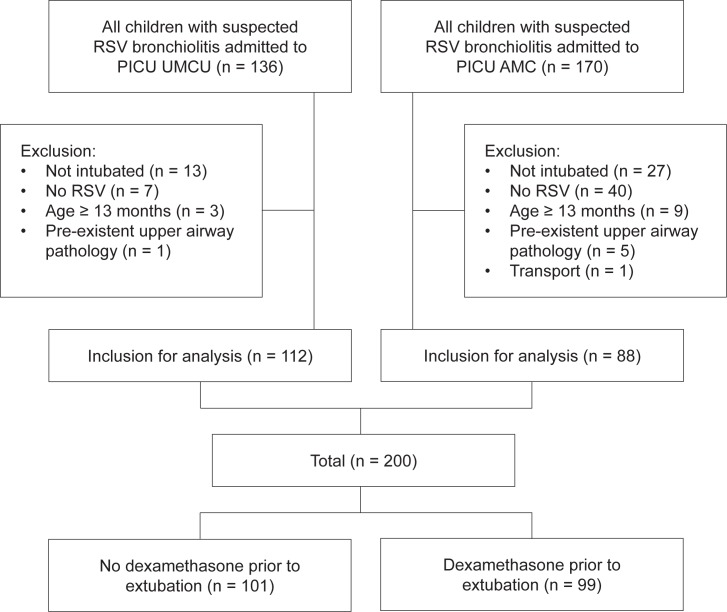
Study flowchart showing the flow of participants through each stage in two PICUs.

Out of 306 eligible patients, 200 patients met the inclusion and exclusion criteria for this study: 112 patients (56%) from the UMCU and 88 patients (44%) from the AMC (Fig 1). Approximately half of the patients received dexamethasone prior to extubation (99 patients, 50%). Dexamethasone administration rates did not differ between the 2 centers (49% vs 50%, p = 0.9). All data were complete, except for exact gestational age in 76 full-term born infants. Tube size of the endotracheal tube at the time of extubation was deemed adequate for weight and age in all patients by 2 authors (ESV and MPH), blinded for the outcome at that time.

Patient and ICU admission characteristics between study groups are shown in Table 1, left panel. Variables were considered to be imbalanced between study groups when the absolute standardized differences were greater than 0.20 (Table 2, first column). A total of 17 (8.5%) patients required reintubation of the trachea because of post-extubation stridor. None of the patients developed stridor necessitating intubation 24 hours after extubation. Within the dexamethasone group 2 patients (2%) required reintubation as compared to 15 patients (15%) within the control group not receiving dexamethasone, with a crude absolute risk reduction (ARR) of 13% (95% CI 5.4–20%). One patient that required reintubation within the dexamethasone group was a full-term born infant, admitted with RSV LRTI at the age of 37 days, who received only 1 dose of dexamethasone just 2 hours prior to extubation. Laryngoscopy showed presence of subglottic stenosis due to granulomas. The other patient was a 12 month old ex-premature with chronic lung disease born at 28 weeks, who also received just 1 dose of dexamethasone 2 hours prior to extubation. No laryngoscopy was done in this patient as there was a combined upper and lower airway problem, as well as a suspected delirium. Three patients who were not given prophylactically dexamethasone received non-invasive ventilation after extubation.

**Table 2:** Comparison of the balance achieved by the propensity score matching for different caliper width as well as comparison to data before matching.

	Before matching	Main analysis^a^		Matching sensitivity analysis^a^	
		Absolute standardized differences—0.30 caliper	Absolute standardized differences—0.10 caliper	Absolute standardized differences—0.20 caliper	Absolute standardized differences—0.40 caliper
Sex	0.02	0.08	0.09	0.10	0.09
Weight^b^	0.38	0.02	0.06	0.01	0.00
Age^b^	0.33	0.07	0.10	0.05	0.07
Down syndrome	0.00	0.08	0.14	0.13	0.07
Congenital heart disease	0.00	0.07	0.11	0.11	0.07
Chronic lung disease	0.20	0.00	0.00	0.00	0.00
Prematurity^b^^,^^c^	0.25	0.00	0.01	0.02	0.01
Palivizumab immunization	0.22	0.12	0.05	0.06	0.12
Endotracheal tube size: 3^b^^,c^	0.37	0.05	0.03	0.01	0.03
Endotracheal tube size: 3.5^b^^,^^c^	0.17	0.04	0.03	0.00	0.03
Endotracheal tube size: 4^b^^,^^c^	0.16	0.00	0.00	0.02	0.01
Endotracheal tube size: 4.5^b^^,^^c^	0.09	0.00	0.00	0.00	0.00
Cuffed endotracheal tube^b^^,^^c^	0.08	0.03	0.06	0.01	0.03
Previous intubations^b^	0.23	0.03	0.01	0.00	0.03
Endotracheal tube replacement^b^	0.46	0.04	0.05	0.04	0.07
Traumatic intubation^b^	0.29	0.03	0.09	0.04	0.03
Duration of ventilation^b^	0.53	0.04	0.10	0.07	0.03
Initial intubation in district general hospital^b^	0.30	0.17	0.30	0.22	0.15
Participating center^b^	0.03	0.05	0.03	0.06	0.05
**Absolute risk reduction (95% CI)**^**d**^	**13% (5.4–20%)**	**13% (5.3–21%)**	**12% (5.2–19%)**	**12% (4.9–19%)**	**14% (5.6–22%)**
Matched number of observations (weighted)	NA	173	140	157	175
Matched number of observations (unweighted)	NA	534	435	486	540

NA = Not Applicable

^a^ Cases were matched on the logit of the propensity score, 3:1 nearest neighbor

^b^ Variable was included in the final propensity score model.

^c^ 2-way and 3-way interactions with variables weight and age were also included in the final propensity score model for this variable.

^d^ The estimated average treatment effect (ATE); 95% confidence intervals were calculated using the Abadie-Imbens standard error for matched samples.

Chronic lung disease, congenital heart disease, Down syndrome, previous intubations during previous admissions, palivizumab prophylaxis were not included as matching variables, because of small numbers. The inclusion of the participating center as one of the covariables improved the propensity score, as well as 2-way and 3-way interactions for endotracheal tube size, prematurity, and use of a cuffed endotracheal tube. The c-statistic of the final propensity score model was 0.79. The balance achieved by the propensity score was considered to be optimal for a caliper of 0.30 (Table 2, second column). The propensity matched ARR was very similar to the crude ARR (ARR 13%, 95% CI 5.3–21%). The absolute standardized differences and ARR estimates were similar for other caliper widths (Table 2). Administration of dexamethasone prior to extubation was associated with a significantly (p = 0.0011) decreased risk of reintubation due to post-extubation stridor as compared to patients not receiving prophylactic dexamethasone (Propensity matched ARR: 13%, 95% CI 5.3–21%). The observed Numbers Needed to Treat within this study was 7.7.

## Discussion

This study examined the incidence of post-extubation stridor requiring reintubation and the effectiveness of prophylactic dexamethasone on reintubation rates for post-extubation stridor in infants with RSV-LRTI, the most common cause of infant respiratory failure in PICUs in winter months. We demonstrated a 8.5% reintubation rate because of post-extubation stridor and a 13% decrease in the risk of reintubation in RSV-LRTI infants treated with prophylactic dexamethasone compared to patients not receiving dexamethasone. The observed rate of EF because of post-extubation stridor in our non-treated patients is high (15%), as overall EF rates for all mechanically ventilated pediatric patients range from 5 to 20% [16–18,28]. The high EF rates in our non-treated patients may be explained by their young age, long duration of mechanical ventilation, and possibly ongoing inflammation of upper airways by the virus in infants with RSV bronchiolitis. Moreover, most scientific literature report overall EF rates and not EF exclusively due to post-extubation stridor. The general rate of EF, not exclusively due to post-extubation stridor, in patients with severe acute bronchiolitis was observed in one study and was reported to be 15% in a small study sample of 40 patients [9].

Most studies that examined the effects of corticosteroids on post-extubation stridor and reintubation have been performed in adults. Randomized controlled trials performed in children were small and showed conflicting results of the efficacy of prophylactic dexamethasone to prevent EF [20,22]. Two meta-analyses were published including the same three pediatric studies, 216 children inclusive [12,20–23]. Of these 2 meta-analyses, the meta-analysis by Mc Caffrey, included one extra pediatric study in children with croup [12,29]. The contradictory and non-significant results may be explained by the heterogeneity (age and subsequent airway diameter and inclusion or exclusion of children with upper airway abnormalities) and different outcomes used in these studies. In contrast to our study population, there was variation in age, disease, definition of EF and the use of dexamethasone (dose and regimen).

A recent study published after these meta-analyses in 124 children older than 3 months found reduced incidence of post-extubation stridor (absolute risk reduction of 17%) in children receiving dexamethasone 0.5 mg/kg/dose during 24 hours compared to children receiving dexamethasone 6 hours prior to extubation. No statistically significant reduction in reintubation rate was found [30]. These studies suggest that evidence for beneficial effect of prophylactically administered corticosteroids still is questioned, not only in infants with RSV LRTI but also in the general pediatric population.

Although the present study would suggest to make infants with acute bronchiolitis the target of a RCT, the non-conclusive results within current literature would warrant a RCT that also includes older and non-bronchiolitis children. However, the RCT should have sufficient power to study the efficacy of prophylactic dexamethasone for different age groups and different ICU admission diagnoses.

There are some limitations to our study. First, the present study is a retrospective observational study and could therefore be subject to residual confounding. Propensity score matching was used to adjust for confounding. The observed effect size within this study is very large, despite adjustment for the most important confounders. Nonetheless, adjustment for unknown confounders is not possible within an observational study [25]. Second, we did not report on side effects of dexamethasone. In retrospective studies, data collection depends on the accuracy of medical records. Possible side effects of dexamethasone would be glucosuria or hypertension, which were not reported systematically in the medical notes. Given the used dosage and brief duration, side effects are not likely to have occurred [30]. Third, there was some variation in dose and regimen of dexamethasone use. Standard prophylactic dexamethasone dose in children in the included centra is 0.15–0.5 mg/kg/dose given 6 hourly. In our study the number of administered doses varied, leading to variation in total dose of 0.15–2 mg/kg/day. The time to start dexamethasone prior to extubation varied from one to 24 hours. Finally, the clinical assessment of stridor is modestly reliable, when comparing assessment by nurses, respiratory therapists and physicians [31]. We tried to minimize this subjectivity by assessing the patient independently by two physicians and by clearly defining the outcome. There are also strengths to our study. We considered infants with RSV LRTI to be as homogeneous as possible in age (up to and including 12 months), diagnosis (RSV LRTI), reason for mechanical ventilation (respiratory failure) and duration of ventilation. Moreover, the study is performed in multi-center setting in two PICUs with a large study population of 200 patients. The study population and the use dexamethasone were comparable between these centers.

## Conclusions

Reintubation due to post-extubation stridor is an important complication of invasive mechanical ventilation for RSV bronchiolitis. Our results suggest a probable beneficial effect of dexamethasone administration prior to extubation. A placebo-controlled trial is needed to confirm the beneficial effect of prophylactic dexamethasone.
